# Thiazolidinedione-8 Alters Symbiotic Relationship in *C. albicans-S. mutans* Dual Species Biofilm

**DOI:** 10.3389/fmicb.2016.00140

**Published:** 2016-02-10

**Authors:** Mark Feldman, Isaac Ginsburg, Abed Al-Quntar, Doron Steinberg

**Affiliations:** ^1^Biofilm Research Laboratory, Institute of Dental Sciences, Faculty of Dental Medicine, The Hebrew University of JerusalemJerusalem, Israel; ^2^Institute of Dental Sciences, Faculty of Dental Medicine, The Hebrew University of JerusalemJerusalem, Israel; ^3^Institute of Drug Research, School of Pharmacy, The Hebrew University of JerusalemJerusalem, Israel

**Keywords:** *C. albicans*, *S. mutans*, S-8, multispecies biofilm, symbiosis

## Abstract

The small molecule, thiazolidinedione-8 (S-8) was shown to impair biofilm formation of various microbial pathogens, including the fungus *Candida albicans* and *Streptococcus mutans*. Previously, we have evaluated the specific molecular mode of S-8 action against *C. albicans* biofilm-associated pathogenicity. In this study we investigated the influence of S-8 on dual species, *C. albicans-S. mutans* biofilm. We show that in the presence of S-8 a reduction of the co-species biofilm formation occurred with a major effect on *C. albicans*. Biofilm biomass and exopolysaccharide (EPS) production were significantly reduced by S-8. Moreover, the agent caused oxidative stress associated with a strong induction of reactive oxygen species and hydrogen peroxide uptake inhibition by a mixed biofilm. In addition, S-8 altered symbiotic relationship between these species by a complex mechanism. Streptococcal genes associated with quorum sensing (QS) (*comDE* and *luxS*), EPS production (*gtfBCD* and *gbpB*), as well as genes related to protection against oxidative stress (*nox* and *sodA*) were markedly upregulated by S-8. In contrast, fungal genes related to hyphae formation (*hwp1*), adhesion (*als3*), hydrophobicity (*csh1*), and oxidative stress response (*sod1*, *sod2*, and *cat1*) were downregulated in the presence of S-8. In addition, *ywp1* gene associated with yeast form of *C. albicans* was induced by S-8, which is correlated with appearance of mostly yeast cells in S-8 treated dual species biofilms. We concluded that S-8 disturbs symbiotic balance between *C. albicans* and *S. mutans* in dual species biofilm.

## Introduction

Multi-species biofilm communities are abundant in the human body. These multi microbial communities play both beneficial and detrimental roles in host homeostasis. As part of a mutual microenvironments, these microorganisms cross-communicate with each other, adjust their population density accordingly, and continuously adapt to the external and internal changes in the biofilm by altering gene expression patterns.

The oral cavity is colonized by numerous of different microbial species. The majority of those microbes reside in biofilms which are attached to variety of oral surfaces. Because of its multispecies nature, the oral microbial community is one of the best biofilm models for the investigation of interspecies interactions (Kolenbrander, 2000).

The yeast *Candida albicans* is a commensal microorganism found in the oral cavity, as well as in other sites of the human body. It is the most common fungal pathogen in humans, causing both mucosal, and systemic infections (Pfaller and Diekema, 2007). Biofilm formation is an important factor in *C. albicans* pathogenesis which involves attachment, colonization, and the development of a mature structure composed of yeast, pseudo- and true hyphae, and extracellular matrix (Finkel and Mitchell, 2011).

*Streptococcus mutans* is an opportunistic pathogen inhabiting the human oral cavity (Ajdic et al., 2002). It has long been recognized as one of the major causes of dental caries (Kutsch and Young, 2011) due to its acidogenic and aciduric properties. Its pathogenicity is strongly coupled with biofilm formation. Biofilm formation in *S. mutans* is strongly associated with formation of extracellular polysaccharides (EPSs) synthesized by the bacterial extracellular enzymes glucosyltransferases (GTFs) and fructosyltransferases (FTFs; Koo et al., 2003). Dietary sucrose is the primary substrate of these enzymes (Yamashita et al., 1993). The glucans synthesized by surface-bound GTFs provide binding sites for bacterial colonization, thus promoting the accumulation of micro-organisms on oral surfaces, which contributes to the bulk and further development of the biofilms (Schilling and Bowen, 1992).

Both bacteria and yeasts are often found together *in vivo* in the same ecological niche. Growing evidences suggest that interspecies, and even inter-kingdom, interactions occur within these mixed populations (Buijssen et al., 2007). Such mixed communities have been found in the oral cavity. For example, the presence of *C. albicans* and *S. mutans* have been documented in early childhood caries and on orthodontic appliances (Falsetta et al., 2014). Moreover, mutans streptococci and *C. albicans* can be found together also in deep carious lesions (Vílchez et al., 2010). Since both microorganism share the same oral environment, numerous studies have investigated the potential interactions between oral streptococci and *C. albicans* in regard to the co-species biofilm development (Bamford et al., 2009; Nobbs et al., 2010; Gregoire et al., 2011; Diaz et al., 2012; Metwalli et al., 2013). A strong mutual relation occurs in dual-species biofilms of *S. mutans* and *C. albicans* resulting in increased biofilm mass and cell densities (Falsetta et al., 2014; Koo and Bowen, 2014; Sztajer et al., 2014). One of the mediators for this cross effect is the streptococcal extracellular GTFB enzyme that firmly binds to *C. albicans* cell surface and thereafter modulates virulent cross-kingdom interaction between these microbes (Gregoire et al., 2011; Hwang et al., 2015).

The biofilm community acquires a number of unique properties, which provides to biofilm defense against various stresses and toxins. The production of reactive oxygen species (ROS) is an unavoidable consequence of an aerobic lifestyle. Because of their reactive nature, ROS can cause oxidative damage to different cellular components such as DNA, proteins, lipids. Their overproduction leads to extensive cell damage and as a result, to cell death (Becerra et al., 2006). To protect themselves against the toxic effects of ROS, cells have acquired many protective mechanisms including enzymes functioning by the removal H_2_O_2_ by catalase of or by superoxide dismutase (SOD) (Becerra et al., 2006). However, the disruption by different stimuli of the balance between the levels of oxidants production and the degree of antioxidant defenses, may lead to a failure to remove the free radicals and consequently seriously impair biofilm development and maintenance (Sardesai, 1995; Arce Miranda et al., 2011).

Various substances were found to affect oral bacterial multispecies biofilm formation (Blanc et al., 2014; Furiga et al., 2014; Wang et al., 2015; Zhang et al., 2015), while only a few were tested against *C. albicans*-bacteria polymicrobial oral biofilm (Müller et al., 2007; Sampaio et al., 2009). Our previous reports demonstrated a pronounced antibiofilm effect of the small molecule, thiazolidinedione derivative, 5-octylidenethiazolidine-2,4-dione (S-8), applied either in solution, or being incorporated into sustained release membrane toward *C. albicans* (Feldman et al., 2014, 2015; Kagan et al., 2014). This molecule has been found to be effective against several types of biofilm (Brackman et al., 2014), including those associated with *C. albicans* (Feldman et al., 2014, 2015). Moreover, as a quorum-sensing quencher, S-8 has the efficiency *in vitro* to prevent catheter-associated urinary tract infections (Shenderovich et al., 2015).

In a recent study we investigated the ability of S-8 to affect *C. albicans-S. mutans* dual species biofilm. In this study we explored the capacity of S-8 to influence biofilm-associated determinants of both microbes, such as EPS production, structure, and morphology, expression of the specific biofilm-related genes and oxidative stress adaptation.

## Materials and Methods

### Synthesis of S-8

The compound S-8 was synthesized in our laboratory and was characterized by Nuclear Magnetic Resonance (NMR) analysis, melting point, and elemental analysis using the same procedure as described previously (Feldman et al., 2014). The purity of the compound was above 95%.

### Mixed Biofilm Formation

The assay was performed as described previously (Sztajer et al., 2014) with minor modifications. Briefly, pre-cultures of *S. mutans* UA159 and *C. albicans* SC5314 were inoculated from single colony grown for 18 h and harvested by centrifugation (5000 r.p.m., 10 min, 4°C). Next, both microbial cultures were diluted to OD_600_ = 0.1 in RPMI + 1% sucrose. The growth medium was supplemented with S-8 at concentrations of 8 and 16 μg/ml. In some experiments Tryptic Soy Broth (TSB) + 1% sucrose medium was used. Equal volumes of each strain (0.1 ml) were inoculated into 96-well microtitre plates. Wells containing mixed cultures without S-8 served as a control. Biofilms were allowed to develop for 48 h, at 5% CO_2_ at 37°C. Biofilm formation was monitored by quantitative PCR.

### Quantification of Mixed Biofilm Biomass Using DNA Quantification

Extraction and quantification of DNA was performed as described previously (Periasamy and Kolenbrander, 2009). Briefly, formed biofilms were washed with PBS. Next, 160 mL 0.05 M NaOH (BioLab Ltd., Jerusalem, Israel) and 40 mL water (DEPC treated; Bio Basic Canada Inc, Markham, ON, Canada) were added to each well. The plates were immersed in a water bath at 60°C for 1 h, and 18.5 μL Tris buffer, pH 7.0 (Eastman Kodak Company, Rochester, NY, USA), was added to each well after the heating period. The extracted DNA samples were stored at -20°C until use. The DNA samples from the mixed biofilms were quantified by means of a quantitative PCR reaction with specific primers for *S. mutans 16S rRNA* and *C. albicans 18S rRNA* using a ABI Prism 7300 (Applied Biosystems, Foster City, CA, USA). The amount of DNA was quantified according to a specific standard curve. The bacterial and fungal genomic DNA for the standard curve analysis was extracted from an overnight culture of *S. mutans* UA159 and *C. albicans* SC5314, respectively, using GenElute Bacterial genomic DNA kit and Plant/Fungi DNA Isolation Kit, respectively (Sigma–Aldrich, St. Louis, MO, USA) according to the manufacturer’s instructions. The genomic DNA was stored at -20°C. The data are presented as a percentage of total biomass in mixed biofilms treated with S-8 and compared to untreated controls. The biomass of each microbe was presented as percentage of the total biomass in each sample. Three separate experiments were performed.

### Confocal Laser Scanning Microscopy (CLSM) of Extracellular Polysaccharides in Mixed Biofilms

Dual species biofilms treated with S-8 at 8 and 16 μg/ml or untreated controls were incubated in 96-well microtitre plates under the conditions described above. For labeling *S. mutans* EPS, 1 mM AlexaFluor 555-labeled dextran conjugate (10,000 MW, Molecular Probes Inc., Eugene, OR, USA) was added to each well before biofilm formation, as described previously (Assaf et al., 2015). Forty-eight hour-biofilms were washed with PBS and incubated for 45 min in PBS containing the fluorescent stain concanavalin A-Alexa Fluor 647 conjugate (ConA; 25 mg/ml) (Invitrogen, Carsbad, CA, USA). ConA (excitation wavelength 650 nm and emission at 668 nm) binds to the glucose and mannose residues of fungal cell wall exopolysaccharides (EPS) (Chandra et al., 2001), and fluoresces red. Stained EPS (blue color for bacteria and red color for fungi) were observed with a Zeiss LSM510 CLS microscope (Carl Zeiss, Oberkochen, Germany). Three-dimensional images of the formed biofilms and EPS distribution were constructed using Zen 2009 software (Carl Zeiss). At least three random fields were observed and analyzed. Three independent experiments were performed. The amount of individual EPS production by *S. mutans* and *C. albicans* in each sample was calculated as blue and red fluorescence intensity, respectively, using Image J v3.91 software (http://rsb.info.nih.gov/ij). The data were presented as individual EPS production by *C. albicans* and *S. mutans* cells in each layer of biofilm (10 μm). The percentage of total EPS production by mixed biofilms treated with S-8 at 8 μg/ml and 16 μg/ml was calculated as area under the curve (AUC) and compared to untreated control. The impact of each individual microbial EPS was calculated as AUC and compared to EPS production within mixed biofilm in each sample.

### Morphology of Mixed Biofilms

After biofilm formation, the wells were washed with PBS, followed by fixation in 4% formaldehyde for 1 h at room temperature. The morphology of bacteria and fungi in mixed biofilms untreated, and treated with 8 and 16 μg/ml of S-8 was then visualized using an analytical Quanta 200 Environmental High Resolution Scanning Electron Microscope (EHRSEM) (FEI, Eindhoven, The Netherlands) at 5,000X magnification. At least four random fields were observed and analyzed. Three independent experiments were performed.

### Quantitative Real Time RT-PCR Analysis *S. mutans* and *C. albicans* Specific Genes in Mixed Biofilm

The assay was performed similarly to that described previously (Feldman et al., 2014). Briefly, dual species biofilms were grown in the absence or presence of S-8 at 8 and 16 μg/ml in 6-well plates under the conditions mentioned above. After washing with PBS, biofilm cells were removed from the bottom of the plates with a sterile scraper following disruption in a Fast Prep Cell Disrupter (Bio 101, Savant Instruments, Inc., NY, USA). Total RNA was extracted from mixed biofilms using Tri-Reagent (Sigma–Aldrich). RNA concentration was determined spectrophotometrically using a Nanodrop ND-1000 Instrument (Wilmington, DE, USA). Two micro gram of template was reverse-transcribed with Super Script First Strand (Invitrogen, Life Technologies, Carlsbad, CA, USA). The integrity and purity of the RNA was assessed using an Agilent 2100 Bioanalyzer system (Agilent Technologies, Santa Clara, CA, USA). Expression of *C. albicans* hyphal/yeast-specific, adhesion and hydrophobicity-associated genes, as well as oxidative stress relative genes (*hwp1, ywp1, als3, csh1, sod1, sod2, cat1*) and *S. mutans* biofilm, stress, and QS-associated genes (*gtfB, gtfC, gtfD, gbpB, brpA, spaA, groEL, nox, sodA, comDE, luxS*) were analyzed. The relative expression levels of the target genes were analyzed using an ABI-Prism 7300 Instrument (Applied Biosystems, Foster City, CA, USA). Platinum SYBR Green PCR Master Mix (Invitrogen) was used to monitor the amplified product in real time, following the manufacturer’s protocol. Primers for the tested genes are listed in **Supplementary Table S1**. For each set of primers, a standard amplification curve (critical threshold cycle vs. exponential of concentration) was plotted, and only those with slope ≈-3 were considered reliable. The PCR involved denaturation at 95°C for 10 min, followed by 40 cycles of amplification (95°C for 10 s, 55°C for 10 s, and 72°C for 10 s) and quantification. The expression of *18S rRNA* and *16S rRNA* was used for normalization and to calculate the relative changes in target gene expression of *C. albicans* and *S. mutans*, respectively. Control reactions were also performed with RNA that had not been reverse-transcribed to ensure that no genomic DNA was amplified during the PCRs. Gene expression is expressed in relative values, setting the expression level of the untreated with S-8 control to 1 for each gene. The assays were performed in triplicate and repeated three times.

### Tetrazolium Reduction Assay

The assay measuring the cell proliferation rate was performed as described previously (Jahn et al., 1995; Hahnel et al., 2012) with some modifications. Briefly, dual species biofilms of *C. albicans* and *S. mutans* formed in 96-well plate with or without added S-8 were washed three times with sterile PBS and overlaid with 100 mM 3-(4,5-dimethyl-2-thiazolyl)-2,5-diphenyl-2H-tetrazolium bromide (MTT). To determine planktonic cell viability, 50 μl of the MTT solution (100 mM) was added to the supernatants aspirated from the biofilms and placed in a new 96-well plate. Plates containing biofilms as well as supernatants were then incubated for 1 h at 37°C. Under these conditions, the lightly yellowish MTT will be reduced to an insoluble blue tetrazolium salt accumulated within the biofilms. The amount of intracellular tetrazolium salts was quantified spectrophotometrically by measuring the absorbance of the solution at 570 nm. The accumulation of tetrazolium salt by the reduction of MTT by cellular dehydrogenases is proportional to the number of viable cells growing in biofilm and planktonic condition. Four independent experiments were performed.

### Oxidative Stress Monitoring in Mixed Biofilms

#### Luminol Dependent Chemoluminescence Assay

Mixed biofilms treated or untreated with S-8 were washed in Hank’s balanced salt solution (HBSS) and re suspended in HBSS and adjusted to the same optical density. The oxidant scavenging abilities (OSA) of co-species biofilms were determined as followed : To 750 μl of HBSS, were added in a sequence, microbial suspensions, luminol (10 mM), H_2_O_2_ (1 mM), sodium selenite (IV) (2 mM), and CoCl_2_.6H_2_O (II) (10 μM). The luminescence generated by this cocktail is due to peroxide and hydroxyl radicals (Ginsburg et al., 2004). Light quenching induced either by untreated or by biofilm cells treated by S-8 at 8 and 16 μg/ml indicated their relative OSA. The data are presented as kinetic curve of the luminescence generation in S-8-treated samples compared to untreated control. Three independent experiments were performed.

#### The Thurman Assay for Measuring H_2_O_2_

Mixed biofilms treated or untreated with S-8 were washed in HBSS and re-suspended in HBSS to an equal optical density. Various amounts of co-species biofilm suspensions were incubated for 5 min with H_2_O_2_ (100 μM). This was followed 1 min by the addition of 20 μl of ferrous ammonium sulfate (10 mM), trichloroacetic acid (TCA) (15 μl from a 30% solution) and potassium thiocyanate (500 μM). Five minutes later, the reaction mixtures were centrifuged at 500 rpm (17 × *g* relative centrifugal force) for 5 min and the red color developed was read at 480 nm in a Cecil 1011 spectrophotometer (Cecil, London, UK) (Thurman et al., 1972). A dose-dependent peroxide curve was also prepared. The uptake of H_2_O_2_ by biofilms were presented as the amounts of H_2_O_2_ remaining un scavenged in each sample treated with S-8, compared to untreated control. To determine the amount of H_2_O_2_ generated by mixed biofilms the supernatants from the above biofilms were mixed with the same reagents, but in the absence H_2_O_2._ The quantity of H_2_O_2_ generated was measured by the Turman assay. The release of H_2_O_2_ from biofilms is presented as H_2_O_2_ (nM) in each supernatant from the S-8-treated biofims, compared to supernatants from untreated control. Three experiments were performed.

#### Detection of ROS

Mixed biofilms untreated or treated with combinations of S-8 and 20 mM N, N’ Diethyldithiocarbamate (DDC) or with 10 mM ascorbic acid (AA) were formed in 96-well black microtitre plates grown for 48 h. Following the washing step with HBSS, endogenous ROS accumulation in biofilms was measured by a fluorometric assay using 10 μM of 2′,7′-dichlorofluorescein diacetate (DCFHDA) (Sigma–Aldrich) (Bink et al., 2011). DCFHDA passively diffuses through the cell membrane into the cell and is deacetylated by esterases to form non-fluorescent 2,7-dichlorofluor-escein(DCFH). The DCFH reacts with ROS to form the fluorescent product 2,7-dichlorofluorescein (DCF) (Fletcher, 1998), which is trapped inside the cell inducing fluorescent. The fluorescence intensities (FIs) of the biofilms were measured with a Tecan plate reader (excitation, 485 nm, emission, 535 nm) for 1 h under shaking at 37°C. The FI values were normalized to the amount of viable cells in biofilms assessed by MTT assay above. Three independent experiments were performed.

## Statistical Analysis

Means of three independent experiments were calculated. The statistical analysis was performed using Student’s *t*-test with a significance level of *P* < 0.05 as compared to controls.

## Results

### S-8 Inhibits Dual Species Biofilm Formation and Modifies Its Microbial Composition

**FIGURE 1:**
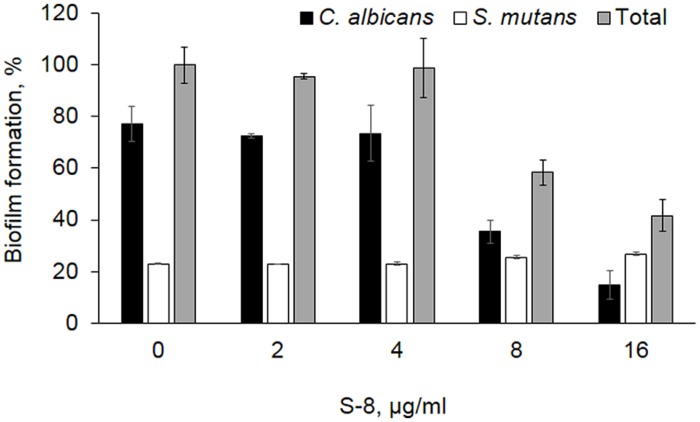
**Effect of S-8 on co-species biofilm formation.** *Candida albicans* –*S. mutans* co-cultures were incubated with S-8 between 2 and 16 μg/ml for 48 h. DNA was extracted from each sample biofilm and quantified by means of qPCR reaction with specific primers for *S. mutans 16S rRNA* and *C. albicans 18S rRNA*. The data are presented as a percentage of total biomass of the mixed biofilms treated with S-8 and compared to untreated control. The biomass of each microbe is presented as percentage of total biomass of each sample. Three independent experiments were performed.

qPCR analysis demonstratedan inhibitory effect of S-8 on total mixed biofilm formation o*f S. mutans* and *C. albicans*. No change in biofilm development was observed using up to 4 μg/ml of S-8. However, increasing S-8 concentration to 8 μg/ml and 16 μg/ml resulted in biofilm inhibition by 42 and 59%, respectively, as compared to the untreated control (**Figure 1**). Data obtained by the MTT assay demonstrated results similar to qPCR dose-dependent inhibition of dual species biofilm by S-8 (data not shown). The influence of S-8 on each individual specie within the mixed biofilm was differed. The relative effect of S-8 on *S. mutans* within the mixed biofilm was negligible at all tested doses as compared to the untreated co-species biofilm (**Figure 1**). In contrast, the portion Candida in the mixed biofilm was dramatically reduced by 55 and 81% under S-8 treatment with 8 and 16 μg/ml, respectively, as compared to *C. albicans* fraction from untreated co-species biofilm (**Figure 1**).

### S-8 Modifies EPS Composition in Mixed Biofilm

**FIGURE 2:**
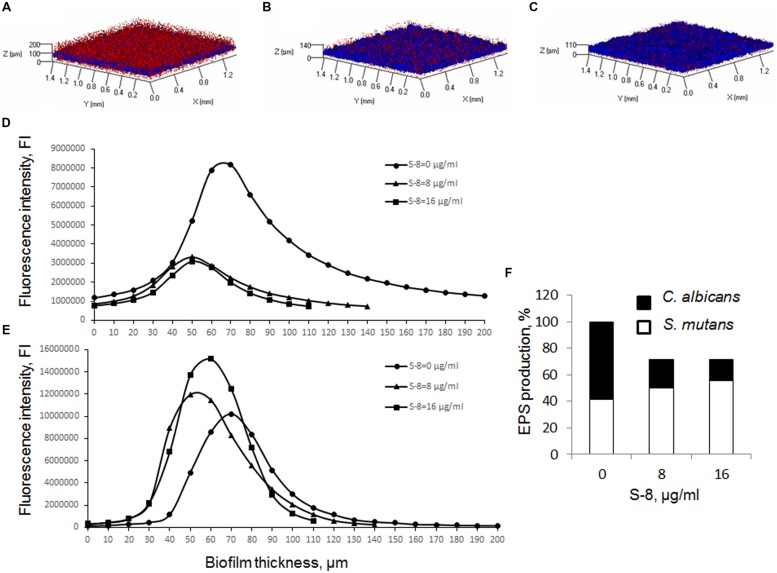
**Effect of S-8 on extracellular polysaccharides (EPS) production in mixed biofilm.** **(A–C)**: CLSM images of EPS produced by untreated biofilms **(A)**, and treated with 8 **(B)** and 16 μg/ml **(C)** of S-8. Stained EPS is presented as blue color for bacteria and red color for fungi. *Z*-axis of three-dimensional constructed images indicates biofilm thickness. Magnification: 10X. At least three random fields were observed and analyzed. Three independent experiments were performed. **(D–F)**: Quantitative measurement of EPS production. The data are presented as individual EPS production by *C. albicans* **(D)** and *S. mutans* **(E)** in each layer of mixed biofilm (10 μm). The percentage of total EPS production by mixed biofilms treated with S-8 at 8 μg/ml and 16 μg/ml is calculated as area under the curve (AUC). The impact of each individual microbial EPS is calculated as AUC and compared to EPS production within mixed biofilm in each sample **(F)**.

Three-dimensional images constructed using CLSM, demonstrated alteration in *C. albicans/S. mutans* mixed biofilm structure under S-8 treatment. The thickness of the co-species biofilm was reduced dose-dependently, by 60 μm (30%) at 8 μg/ml of S-8 (**Figure 2B**), and by 90 μm (45%) at 16 μg/ml (**Figure 2C**), as compared to controls (**Figure 2A**). Within the biofilm structure, total EPS formation was reduced by 29% in the presence of S-8 at both tested doses of 8 and 16 μg/ml as compared to control (**Figure 2F**). However, the pattern of EPS production by each individual microbe calculated as AUC for *C. albicans* (**Figure 2D**) and *S. mutans* (**Figure 2E**) due to S-8 treatment varied. Untreated mixed biofilm consisted of slightly higher amounts of fungal EPS (58%), compared to bacterial EPS (42%) (**Figures 2A,F**). However, exposure to 8 μg/ml of S-8 caused a substantial change in EPS composition in co-species biofilm: a majority of *S. mutans* EPS (71%) and minority of EPS of *C. albicans* (29%) (**Figures 2B,F**). S-8 at a concentration of 16 μg/ml further modified EPS impact in favor of *S. mutans* (78%), while fungal polysaccharides consisted only 22% of total EPS (**Figures 2C,F**).

### S-8 Affects Morphology of the Mixed Biofilm

**FIGURE 3:**
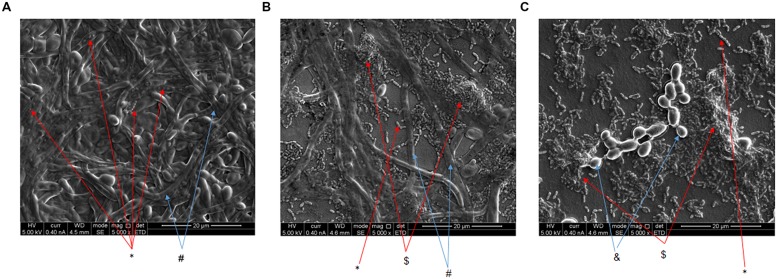
**Morphology of mixed biofilms.** Co-species biofilms formed in the presence or absence of S-8 were fixed with formaldehyde. Morphology of the *C. albicans* and *S. mutans* in mixed biofilms was then visualized by scanning electron microscopy. **(A)** Untreated control. **(B,C)** Biofilms developed with 8 and 16 μg/ml of S-8, respectively. Red arrows indicate streptococci cells (^∗^) or aggregated ($) streptococci cells. Blue arrows indicate *C. albicans* yeast (&) or hyphae cells (#). Magnification: 5,000X. At least three random fields were observed and analyzed. Three independent experiments were performed.

Microscopic observation showed that S-8 dramatically alters biofilm morphologic composition. As shown in **Figure 3**, untreated control biofilm (**Figure 3A**) consisted predominantly of candidal branched hyphae (#, blue arrow) and characterized by highly dense mycelium, while streptococcal cells (^∗^, red arrow) are spread across fungal filaments. However, S-8 already at 8 μg/ml influenced bacterial and fungal morphology. *S. mutans* are seen aggregated in clusters ($, red arrow), while density of fungal mycelium decreased (**Figure 3B**). Furthermore, S-8 at dose of 16 μg/ml leads to the alteration of yeast–to-hyphae transition resulting in the appearance of mainly yeast form of *C. albicans* (&, blue arrow) (**Figure 3C**). Bacteria appeared mostly attached to each other forming aggregates ($, red arrow) (**Figure 3C**).

### Effect of S-8 on the Genes Expression in Dual Species Biofilm

**FIGURE 4:**
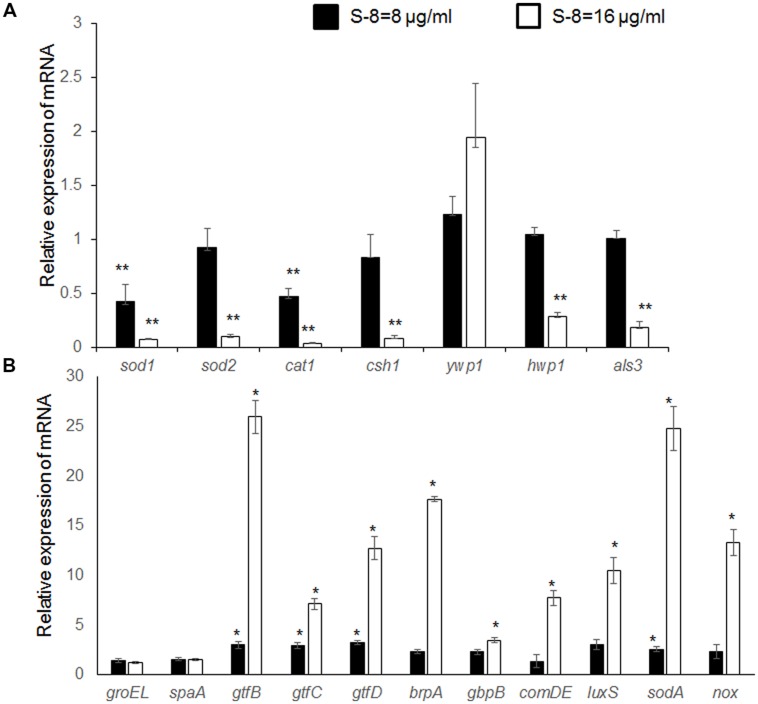
**Quantitative real time RT-PCR analysis of *C. albicans* and *S. mutans* specific genes.** Mixed biofilms were formed in the presence or absence of 8 and 16 μg/ml S-8 and expression of the target genes was determined by quantitative real-time RT-PCR. Housekeeping genes *C. albicans 18S rRNA* and *S. mutans 16S rRNA* were used for normalization. **(A)** Candidal specific genes. **(B)** Streptococcal specific genes. The expression level of the untreated sample is set to one for each gene.^∗^Significantly up regulated than untreated control (*P* < 0.05). ^∗∗^Significantly down regulated than the untreated control (*P* < 0.05). The assays were performed in triplicate and the means ± SD from three independent experiments were calculated.

To evaluate the mechanisms underlying S-8’s effect on biofilm formation and composition, we analyzed changes in *C. albicans* and *S. mutans* gene expression levels in mixed biofilms exposed to S-8 (**Figure 4**). We investigated fungal genes which are essential in biofilm development including those associated with adhesion and hyphal formation (*als3* and *hwp1*), cell surface hydrophobicity (*csh*1), and yeast wall protein (*ywp*1). The transcriptional levels of genes involved in the adhesion process, such as the hyphal-specific genes *hwp1* and *als3*, were significantly downregulated by S-8 at 16 μg/ml by 3.5- and 5.4-fold, respectively, as compared to untreated control (**Figure 4A**). CSH plays an important role during biofilm formation in *Candida albicans*. The *csh1* gene, which codes for the CSH-associated protein, is the first candidate gene that has been demonstrated to play a role in affecting the CSH phenotype in *C. albicans*. S-8 at 8 and 16 μg/ml dose-dependently reduced *csh1* expression in mixed biofilm by 1.2- and 12.5-fold, respectively, as compared to control (**Figure 4A**). The transcription of *ywp*1 gene, regarded as a specific marker for the yeast form of *C. albicans* and also as an inhibitor of adhesion activities, was upregulated twofold by S-8 at 16 μg/ml, as compared to control (**Figure 4A**).

In parallel, we tested biofilm-associated genes of *S. mutans* in mixed biofilm including EPS glucan formation genes (*gtfB*, *gtfC*, *gtfD*), glucan-binding protein (*gbpB*), cell surface antigen (*spaA*), surface-associated protein (*brpA*), QS-associated genes (*comDE*, *luxS*) and stress-responsive proteins (*groEL, sodA, nox*). All three tested *gtf*s genes were dramatically upregulated in a dose-dependent manner by S-8 in the co-spcies biofilm. Briefly, the presence of 16 μg/ml of S-8 in mixed biofilm, increased mRNA transcription of *gtfB* by 26-fold, *gtfC* by sevenfold, and *gtfD* by 13-fold, as compared to untreated control (**Figure 4B**). Moreover, *gbpB* gene, encoding to surface-associated glucan-binding protein, and thereby promoting plaque formation, was also upregulated by 3.5-fold under exposure of S-8 at 16 μg/ml as compared to control (**Figure 4B**). In addition, *brpA* transcript translated to another surface-associated protein playing important role in regulation of biofilm formation, was strongly elevated by 18-fold with in presence of 16 μg/ml of S-8, as compared to control (**Figure 4B**). In contrast, surface antigen *spaA* expression remained unchanged in the presence of S-8 (**Figure 4B**). Furthermore, the agent caused a marked induction of both QS-associated pathways gene expression: *luxS* which synthetizes autoinducer 2 (AI-2) and *comDE* involved in sensing and response regulation of competence stimulating peptide (CSP). A dose-dependent increase by 3- and 10.5-fold of *luxS* transcript was detected following biofilm treatment with S-8 at doses of 8 and 16 μg/ml, respectively, while *comDE* mRNA was increased by 7.7-fold by S-8 at 16 μg/ml, as compared to untreated control (**Figure 4B**).

Our data show a notable induction by S-8 of oxidative stress related genes of *S. mutans*. Streptococcal *nox* and *sodA* genes were upregulated by 2.5-fold, already by 8 μg/ml of S-8, while increasing the dose of the agent up to 16 μg/ml, caused a further significant induction of these genes by 13- and 25-fold, respectively, as compared to control (**Figure 4B**). However, another stress-associated *S. mutans groEL* gene was not affected by S-8 (**Figure 4B**). In contrast to the induction by S-8 of bacterial genes related to oxidative stress response, fungal genes associated with defense against oxidative stress were downregulated in mixed biofilms exposed to the agent. *C. albicans sod1* and *cat1* expressions were decreased by more than twofold at S-8 = 8 μg/ml, while increasing the dose of the agent to 16 μg/ml, caused a drastic downregulation of these genes by 13.5- and 28.5-fold, respectively, as compared to controls (**Figure 4A**). Finally, candidal *sod2* mRNA was reduced by10-fold at 16 μg/ml of S-8, as compared to control (**Figure 4A**).

### Effect of S-8 on OSA, H_2_O_2_ Uptake and Excretion by Mixed Biofilms

**FIGURE 5:**
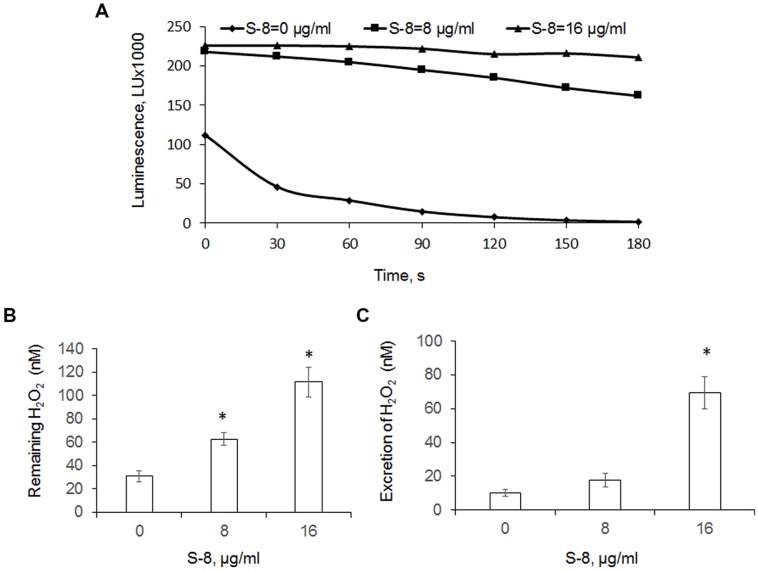
**Oxidative stress response in mixed biofilm.** **(A)** The oxidant scavenging abilities (OSA) of co-species biofilms. Light quenching induced either by untreated or by biofilm cells treated by S-8 at 8 and 16 μg/ml indicated their relative OSA. The data are presented as kinetic curve of the luminescence generation in S-8-treated samples compared to untreated control. Three independent experiments were performed. **(B)** Mixed biofilms treated or untreated with S-8 were incubated for several minutes with H_2_O_2_. The uptake of H_2_O_2_ by biofilms are presented as remaining H_2_O_2_ amount (nM) in each treated with S-8, compared to untreated control. The results are presented as means ± SD of three independent experiments. ^∗^Significantly higher than the value for the untreated control (*P* < 0.05). **(C)** Quantity of secreted hydrogen peroxide was measured in mixed biofilm supernatants. The release of H_2_O_2_ from biofilms is presented as H_2_O_2_ amount (nM) in each supernatant of treated with S-8 biofims, compared to supernatant of untreated control. The results are presented as means ± SD of three independent experiments. ^∗^Significantly higher than the value for the untreated control (*P* < 0.05).

Washed suspensions of *C. albicans*/*S. mutans* biofilms were tested for their ability to scavenge ROS generated by the peroxide cocktail. S-8 untreated biofilms strongly scavenged ROS in a time-dependent mode, while biofilms treated by tested concentrations of S-8 had only minimal scavenging properties (**Figure 5A**). Moreover, initial amount of ROS (time 0) was almost twofold lower in untreated biofilms as compared to biofilms formed in the presence of S-8 (**Figure 5A**). In other assays, biofilms treated with S-8 were mixed with 100 μM of H_2_O_2_ and incubated at room temperature for 5 min and the amounts of unscavenged peroxide were measured by the Thurman reagent (Thurman et al., 1972). Increasing concentration of S-8 prevented the uptake of peroxide by the biofilm (**Figure 5B**). In addition, using Thurman reagent, we observed a marked induction of hydrogen peroxide release by co-species biofilms treated with 16 μg/ml of S-8, as compared to control (**Figure 5C**).

### Endogenous ROS Accumulation by S-8-Treated Biofilms

**FIGURE 6:**
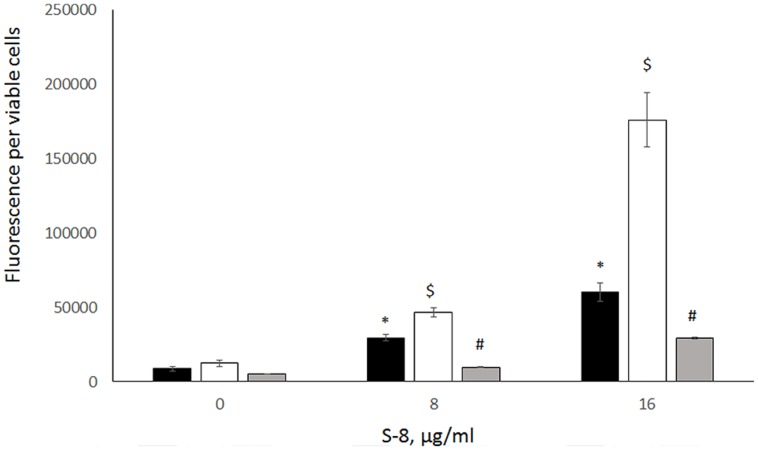
**Reactive oxygen species (ROS) accumulation in mixed biofilms.** Mixed biofilms untreated or treated with combinations of S-8 and N, N′ Diethyldithiocarbamate (DDC) or ascorbic acid (AA) were formed for 48 h. ROS accumulation in biofilms was measured in a fluorometric assay using 2′,7′-dichlorofluorescein diacetate (DCFHDA). The fluorescence intensities (FIs) of the biofilms were measured with a Tecan plate reader (excitation, 485 nm, emission, 535 nm). The FI values were normalized to the amount of viable cells in biofilms. The results are presented as means ± SD of three independent experiments.^∗^Significantly higher than the value for the untreated control (*P* < 0.05). ^$^Significantly higher than the value for the untreated with DDC biofilm (*P* < 0.05). ^#^Significantly lower than the value for the untreated with AA biofilm (*P* < 0.05).

In other experiments, production of endogenous ROS by co-species biofilm was monitored using a fluorometric assay. A dose-dependent increase in ROS accumulation was recorded in biofilms exposed to 8 and 16 μg/ml of S-8 as compared to untreated control (black columns, **Figure 6**). Moreover, co incubation of dual species biofilms with S-8 and a SOD inhibitor, DDC, resulted in increased endogenous ROS levels in mixed biofilms as compared to results with S-8 treatment alone (white columns, **Figure 6**). The most pronounced generation of ROS was detected when mixed biofilms were treated with DDC and the highest tested dose of S-8 = 16 μg/ml. Addition of the antioxidant, AA, significantly reduced ROS accumulation in all untreated and S-8 treated samples (gray columns, **Figure 6**). The MTT assay showed that the viability of biofilm and planktonic cells exposed either to DDC or AA alone or in combination with S-8 was not significantly altered (data not shown).

## Discussion

Previous reports provided a clear evidence that *S. mutans* and *C. albicans* co-existed in a strong synergism in biofilm, thus enhancing their virulence (Falsetta et al., 2014; Koo and Bowen, 2014; Sztajer et al., 2014). In the present study, we showed that S-8, dose-dependently, decreased total biomass of *C. albicans-S. mutans* mixed biofilm. Interestingly, the individual microbe in co-species biofilm behaved differently upon exposure to S-8. In previous studies it was demonstrated that S-8 attenuated biofilms of *C. albicans* and fungal pathogenicity (Feldman et al., 2014, 2015; Kagan et al., 2014). S-8 at concentrations up to 32 μg/ml did not exhibit any significant effect on single *S. mutans* biofilm formation (unpublished data). The *C. albicans* DNA load in mixed biofilm was strongly reduced by S-8 in mixed biofilm. The decrease of fungal fraction was associated with alteration of fungal morphology and down regulation of genes related to fungal cell surface hydrophobicity and adhesion, such as *csh1* and *asl3*, respectively. These findings are in agreement with our previous reports demonstrating that attenuation of above virulence characteristics of *C. albicans* due to S-8 treatment is critical in inhibition of fungal biofilm development (Feldman et al., 2014, 2015). Although in absolute values, streptococcal fraction was not change significantly due to exposure to S-8, the bacteria/fungi ratio was attenuated dramatically in favor of *S. mutans* with increasing concentration of the agent. We proposed that a relative increase in bacterial fraction may lead to induction of both quorum-sensing (QS) pathways, *comDE* and *luxS* of *S. mutans*, observed in our study. That, in turn, could also contribute to inhibition of fungal filamentation. Indeed, it was demonstrated that *S. mutans* suppress the formation of *C. albicans* hyphae in a co-culture model via production of QS molecule, competence-stimulating peptide (CSP) associated with *comDE* gene. (Jarosz et al., 2009). Another QS molecule, autoinducer 2 (AI-2) has been proposed to be a general interspecies signal, and synthesized by LuxS. AI-2, of *Aggregatibacter actinomycetemcomitans* was shown to inhibit *C. albicans* hypha- and biofilm formation (Bachtiar et al., 2014). Thus, we suggested that both QS pathways of *S. mutans* are essential in controlling *C. albicans-S. mutans* mixed biofilm development upon exposure to S-8.

Another intriguing observation was related to EPS production. Total EPS matrix in the mixed biofilm was reduced by S-8. However, polysaccharide synthesis by each microbe was affected by S-8 in dual species biofilm differently. Dramatic change in fungal/bacterial EPS ratio in strong favor to *S. mutans* was detected when treated with S-8 samples. Significant upregulation of streptococcal EPS-associated genes, *gtf*s and *gbpB*, as well as the appearance of bacterial aggregates probably joined with EPS support these results. On other hand, transcript of candidal glucan synthase gene *fks1* was significantly reduced by S-8 (unpublished data) which is correlated with a pronounced decrease of fungal EPS production. Sztajer et al. (2014) have reported that *C. albicans* is more efficient than *S. mutans* in uptake of sucrose. Since GTFs enzymes are induced by their substrate sucrose, *gtf*s transcription was down regulated due to the depletion of this sugar. They have also reported that in the S. *mutans-C. albicans* dual biofilm expression of the glucan-binding protein, GbpC was strongly down regulated. In our system, the presence of S-8 resulted in induction of *S. mutans* EPS-associated genes thereby the amount of bacterial EPS was found to be high as compared to untreated control.

In present study we observed that S-8 had a marked induction of ROS which may cause oxidative stress in dual species biofilm. Oxidative stress is a result of an imbalance between the production of oxidants, such as free radicals, peroxide and nitric oxide, and with the levels of antioxidant defenses. The aerobic growth of *S. mutans* is accompanied by the induction of specific scavengers of the ROS generated during the oxygen metabolism. The activity of key enzymes, such as SOD and NADH oxidase (Nox) undergo a significant enhancement upon exposure of *S. mutans* to oxygen (De Vendittis et al., 2010). The present study showed that the notable accumulation of ROS was associated with a strong up regulation of *S. mutans nox* and *sodA* gene expression in mixed biofilms treated with S-8. It has previously shown that Nox is the major oxygen-metabolizing enzyme used by *S. mutans*, and is responsible for reduction of approximately 40% of the dissolved oxygen encountered in dental plaque, and that the Nox enzyme shows elevated activity under conditions of oxidative stress (Baker et al., 2014). Other study demonstrated that *S. mutans* genes *nox* and *sodA* were strongly induced by H_2_O_2_ (Galvão et al., 2015). In addition, *brpA* gene encoding to cell surface-associated biofilm regulatory protein, have also been shown to play critical roles in environmental stress responses and biofilm development by *S. mutans* (Wen et al., 2006) which was significantly induced by S-8 treatment. Interestingly that the expression of another streptococcal stress-associated gene, *groEL* was not altered by the presence of S-8. This could be explained by the fact that this protein is mostly induced by heat shock and acidic stress (Lemos et al., 2005).

Various antimicrobial molecules are known to induce the accumulation of ROS in *C. albicans* (Aerts et al., 2007; François et al., 2009; Bink et al., 2011) and *S. mutans* (Coenye et al., 2007). In the antioxidant defense system, the main candidal enzymes involved in the scavenging of ROS are SODs and catalase. For instance, occurrence of miconazole-tolerant persisters in *C. albicans* biofilms is associated with ROS-detoxifying activity of SODs (Bink et al., 2011). In addition, *C. albicans* cells were protected from amphotericin B-induced oxidative damage by exogenous catalase and/or SOD (Sokol-Anderson et al., 1986). However, the balance between the production of oxidants and the levels of antioxidant defenses can be significantly affected by different environmental stresses. When this balance is impaired under unfavorable conditions, an increase in the ROS production or a reduction in the antioxidant defenses (or both) may further aggravate the ROS burden on the cells (Becerra et al., 2006; Arce Miranda et al., 2011; Baronetti et al., 2011). Furthermore, our results showed that *C. albicans* in untreated mixed biofilm is rich in catalase capable of scavenging ROS. In contrast, mixed biofilms treated with S-8 markedly prevented scavenging of luminescence by the peroxide cocktail and also the uptake of H_2_O_2_, indicating alteration of *C. albicans* catalase activity and/or synthesis. Although *S. mutans* as a facultative anaerobe lacks catalase and a full electron transport chain, there is an alternative pathway in these bacteria aimed to resist peroxide-associated oxidative stress (Ryan and Kleinberg, 1995; Fujishima et al., 2013). Moreover, the excretion of H_2_O_2_ was dramatically induced by S-8 in co-species biofilm. H_2_O_2_ is released by numerous bacteria (Ryan and Kleinberg, 1995) and serves as an important factor in microbial competition for survival. *C. albicans* is in contact with ROS produced by H_2_O_2_-producing bacteria in the mouth. Several commensal bacteria of the oral cavity as *Enterococcus faecalis* and Lactobacillus species, secrete ROS into their surroundings, and this has an inhibitory effect on the growth of *C. albicans* in host niches (Dantas Ada et al., 2015). In addition, viridans group streptococci inhabiting oral cavities also have an antagonistic effect on many bacterial species by excretion H_2_O_2_ (Barnard and Stinson, 1999). Since *S. mutans*, among other oral streptococci, is considered as an H_2_O_2_ producer (Carlsson et al., 1983), we proposed that significant elevation of H_2_O_2_ induced by S-8 in mixed biofilm could contribute to streptococcal competition with *C. albicans* in the same ecological niche. On the other hand, fungal defense response against oxidative stress, including antioxidant genes (*sod1*, *sod2*, and *cat1*) expression, was strongly attenuated by the S-8. Therefore, we proposed that by induction of ROS along with down regulation of fungal oxidative stress response genes in mixed biofilm, S-8 impairs *C. albicans* protection mechanism against oxidative stress.

Although an antioxidant and ROS scavenger, AA (Bink et al., 2012) significantly decreased the presence of endogenous ROS induced by S-8 in dual species biofilm, it was not able to attenuate biofilm inhibitory activity of S-8. Therefore, it seems that ROS induction by S-8 is a secondary result of S-8 activity and not essential for S-8 anti biofilm activity. In addition, SOD inhibitor DDC markedly enhanced S-8’ ROS-inducing activity. Previous investigations showed the ability of DDC to increase the efficacy of miconazole against *C. albicans* biofilms (Bink et al., 2011) and enhance group A hemolytic streptococci killing by streptolysin S (Ginsburg et al., 1997). Unfortunately, DDC exhibits toxicity toward mammalian cells, and is not suitable for human therapy. However, the combination of specific inhibitors of SOD with no side effects and proven non-toxic S-8 agent (Feldman et al., 2015), might lead to a novel therapy of biofilm-associated infections.

## Conclusion

Our data indicate that in addition to its inhibitory effect on total dual species *C. albicans-S. mutans* biofilm formation, S-8 also alters symbiotic relationship between these two microorganisms. The interactions between fungi and streptococci appear to be essentially synergistic which leads to a strong enhancement of pathogenicity of the co-culture biofilm (Falsetta et al., 2014). The stress-caused competition between fungi and bacteria for nutrients, niche, and survival, disrupts the symbiotic relationship. Therefore, the addition of specific inhibitors of bacterial biofilm, such as natural polyphenols, could be beneficial for enhancing efficiency of S-8 against polymicrobial biofilms.

## Author Contributions

MF and IG contributed to the design of the work, as well as to the acquisition, analysis, and interpretation of data for the work; AA-Q contributed to the synthesis and analysis of S-8; DS contributed to the conception of the work; revising it critically for important intellectual content; final approval of the version to be published.

## Conflict of Interest Statement

The authors declare that the research was conducted in the absence of any commercial or financial relationships that could be construed as a potential conflict of interest.
