# Supplementary material for: Thiazolidinedione-8 Alters Symbiotic Relationship in C. albicans-S. mutans Dual Species Biofilm
Source: Front Microbiol. 2016 Feb 10;7:140. doi: 10.3389/fmicb.2016.00140 (PMC4748032; doi:10.3389/fmicb.2016.00140)
Supplement: Supplementary file 1 [file Table_1.DOCX]

**Supplementary Table S1. Primers used in this study**

|  |  |
| --- | --- |
| ***S. mutans* primer**s | **Sequence (5′–3′)** |
| *gtfB*-F | AGCAATGCAGCCAATCTACAAAT |
| *gtfB*-R | ACGAACTTTGCCGTTATTGTCA |
| *gtfC*-F | CTCAACCAACCGCCACTGTT |
| *gtfC*-R | GGTTTAACGTCAAAATTAGCTGTATTAGC |
| *gtfD*-F | ACAGCAGACAGCAGCCAAGA |
| *gtfD*-R | ACTGGGTTTGCTGCGTTTG |
| *gbpB*-F | ATGGCGGTTATGGACACGTT |
| *gbpB*-R | TTTGGCCACCTTGAACACCT |
| *brpA*-F | GGAGGAGCTGCATCAGGATTC |
| *brpA*-R | AACTCCAGCACATCCAGCAAG |
| *spaA*-F | GACTTTGGTAATGGTTATGCATCAA |
| *spaA*-R | TTTGTATCAGCCGGATCAAGTG |
| *groEL*-F | CCAGGAGCTTTGACTGCGAC |
| *groEL*-R  *nox*-F  *nox*-R  *sodA*-F  *sodA*-R | TTGCGGATGATGATGTAGATGGT  GGGTTGTGGAATGGCACTTTGG  CAATGGCTGTCACTGGCGATTC  GCAGTGCTAAGACTCCCGAATC  TTGCGGAAGTGTGAGATTGGC |
| *16S rRNA*-F | CCTACGGGAGGCAGCAGTAG |
| *16S rRNA*-R | CAACAGAGCTTTACGATCCGAAA |
| ***C. albicans* primers** |  |
| *csh1*-F | CTGTCGGTACTATGAGATTG |
| *csh1*-R | GATGAATAAACCCAACAACT |
| *ywp1*-F | GCTACTGCTACTGGTGCTA |
| *ywp1*-R | AACGGTGGTTTCTTGAC |
| *als3-F* | CAACTTGGGTTATTGAAACAAAAACA |
| *als3-R* | AGAAACAGAAACCCAAGAACAACC |
| *hwp1-F* | GCTCCTGCTCCTGAAATGAC |
| *hwp1-R* | CTGGAGCAATTGGTGAGGTT |
| *18S rRNA-F* | CACGACGGAGTTTCACAAGA |
| *18S rRNA-R*  *sod1-F*  *sod1-R*  *sod2-F*  *sod2-R*  *cat1-F*  *cat1-R* | CGATGGAAGTTTGAGGCAAT  TTGAACAAGAATCCGAATCC  AGCCAATGACACCACAAGCAG  ACCACCCGTGCTACTTTGAAC  GCCCATCCAGAACCTTGAAT  TTACGTTCAAGTTCATTTCATCAG  CAATCTGTGCGGTCTGTGAG |
